# Supplementary material for: Cannabis sativa (Hemp) seed-derived peptides WVYY and PSLPA modulate the Nrf2 signaling pathway in human keratinocytes
Source: PLoS One. 2024 May 23;19(5):e0298487. doi: 10.1371/journal.pone.0298487 (PMC11115254; doi:10.1371/journal.pone.0298487)
Supplement: S1 File — (DOCX) [file pone.0298487.s001.docx]

**Supplementary information**

**Specific Roles of WVYY and PSLPA Peptides in the Nrf2 Signaling Pathway in Human Keratinocytes**

**Euihyun Kim^1^, Jihyeon Jang^1^, Hyo Hyun Seo^1^, Jung Hun Lee^1^, Sang Hyun Moh^1*^**

^1^ Plant cell Research Institute of BIO-FD&C Co. Ltd., Incheon 21990, Korea

*Correspondence :

Sang Hyun Moh, biofdnc@gmail.com; Plant cell Research Institute of BIO-FD&C Co. Ltd., Incheon 21990, Korea

Table S1. Sequence-specific primers used for quantification of differentially expressed transcripts.

| Genes | Primer sequences (5’–3’) | Product size (bp) | Accession No. /  Category No. |
| --- | --- | --- | --- |
| *GAPDH* | Commercialized primer (Qiagen) | - | QT01192646 |
| *Nrf2* | F: GCTGCTCAGAATTGCAGAAA  R: CGTAGCATGCTGAAAACTTCG | 179 | NM_001145412.3 |
| *KEAP1* | F: ATGATGGTCACACGTTCCTG  R: CTGCTGGTCAATCTGCTTCC | 146 | NM_012289.4 |
| *CUL3* | F: CCCAGAGAGGAAAGAAACAAG  R: GTAAGAATCGCGCCTTCAAC | 152 | NM_001257197.2 |
| *HO-1* | F: ATGACACCAAGGACCAGAGC  R: GTGTAAGGACCCATCGGAGA | 153 | NM_002133.3 |
| *CAT* | F: CACTGAGGTCCACCCTGACT  R: CAGATTTGCCTTCTCCCTTG | 136 | NM_001752.4 |
| *SOD1* | F: TGTGGCCGATGTGTCTATTG  R: ACCACAAGCCAAACGACTTC | 163 | NM_000454.5 |
| *SOD2* | F: TTCAATAAGGAACGGGGACA  R: ACACATCAATCCCCAGCAGT | 100 | NM_000636.4 |
| *KGF* | Commercialized primer (Qiagen) | - | QT01194676 |
| *BAX* | F: AGCGACTGATGTCCCTGTCT  R: CTCAGCCCATCTTCTTCCAG | 130 | NM_001291428.2 |
| *BCL2* | F: TGCCTTTGTGGAACTGTACG  R: TCTTCAGAGACAGCCAGGAGA | 68 | NM_000633.3 |

Table S2. Prediction of antioxidant activity of WVYY and PSLPA

| Peptide | Pre-score^a^ | Pre-Activity^b^ | Length | Mass (Da) | pI^c^ | Hydrophilicity^d^ | |
| --- | --- | --- | --- | --- | --- | --- | --- |
| WVYY | 0.8007 | AnOxP^e^ | 4 | 629.7143 | 5.8650 | | 1.0000 |
| PSLPA | 0.9997 | AnOxP | 5 | 483.5490 | 5.9750 | | 0.8000 |
| ^a^ Pre-score: Probability score  ^b^ Pre-activity: Predictive activity.  ^c^ pI: Isoelectric point.  ^d^ Hydrophilicity: Hydrophobic residue ratio.  ^e^ AnOxP = Antioxidative peptide, non-AnOxP = non-Antioxidative peptide. | | | | | | | |
|  | | | | | | | |

| Peptide | Class | Probability |
| --- | --- | --- |
| WVYY | TIP | 0.862 |
| PSLPA | Non-TIP | 0.000 |

Table S3. Prediction of tyrosinase inhibitory peptides (TIP) of WVYY and PSLPA
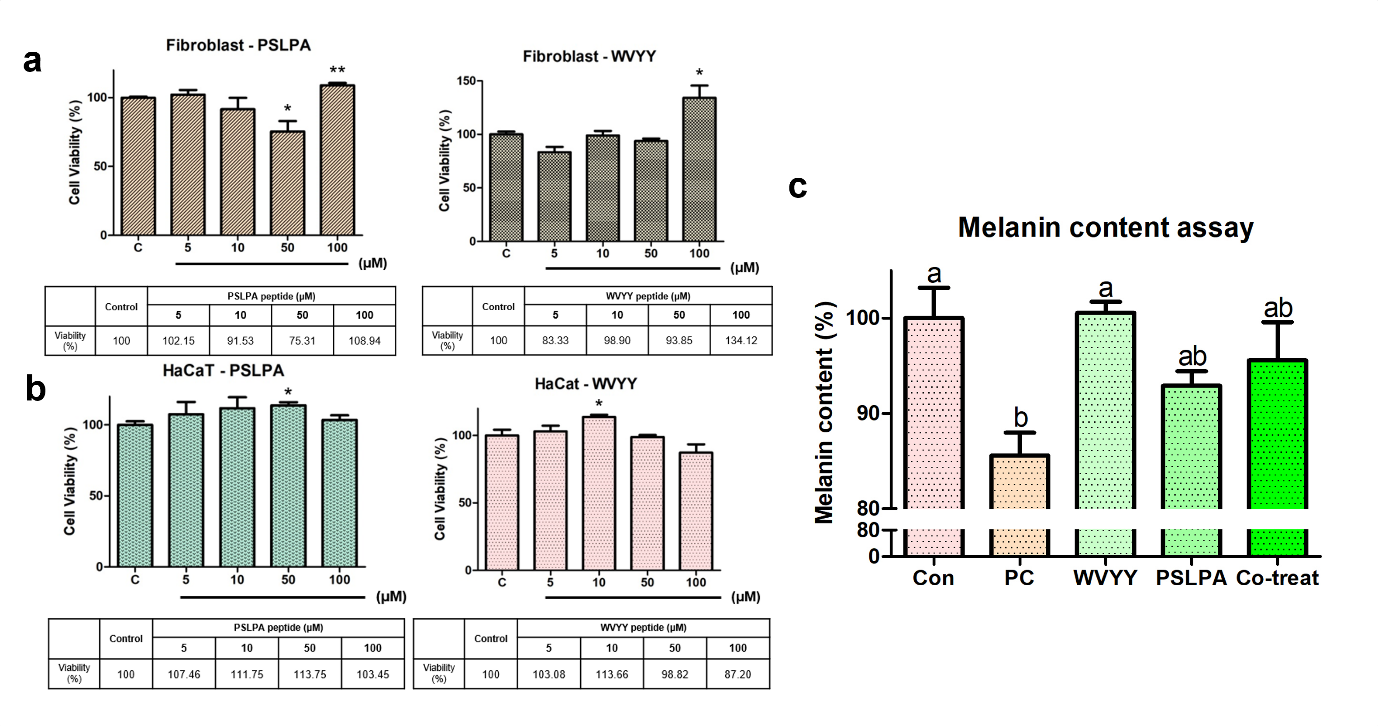
Figure S1. Concentration optimization of WVYY and PSLPA via assessments of cell viability and melanin
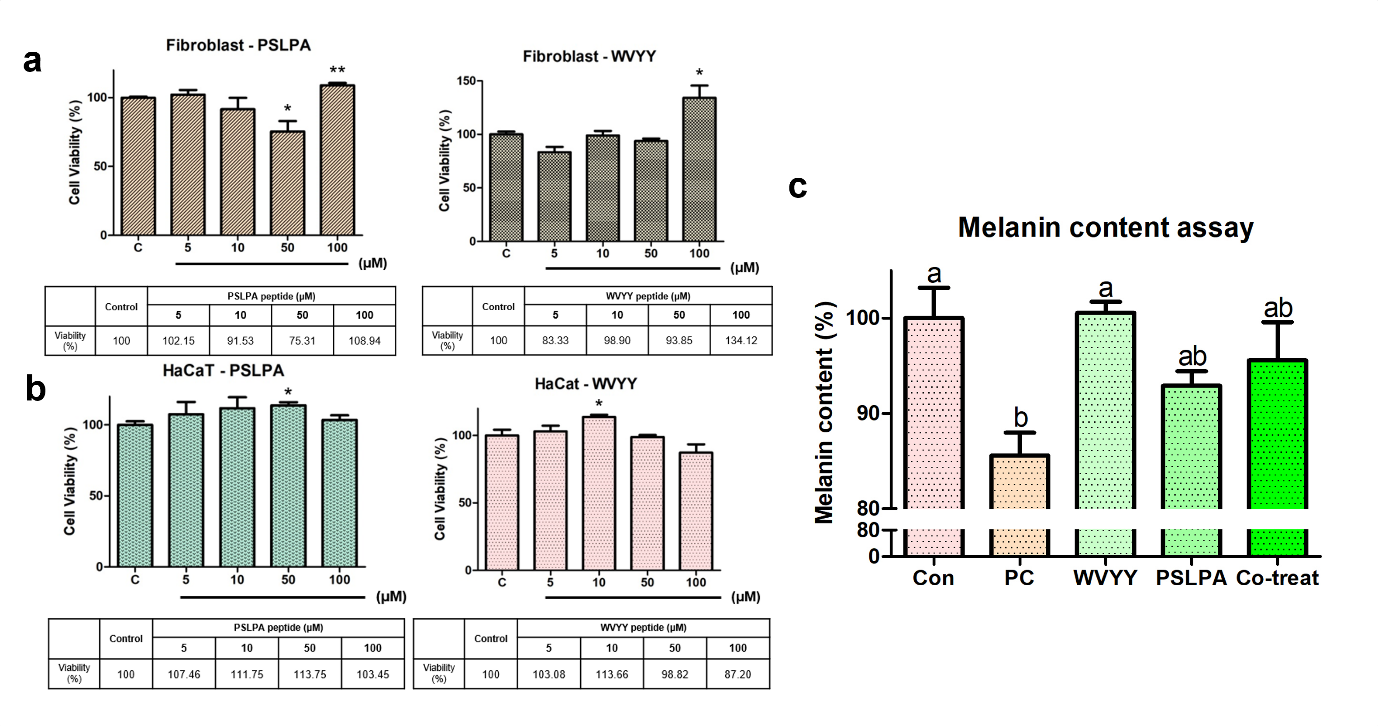
content

Figure S1. Concentration optimization of WVYY and PSLPA and assessment of melanin content. In the initial concentration range, a serial dilution (5, 10, 50, and 100 µM) of WVYY and PSLPA was applied to human keratinocytes (HaCaT cells) and fibroblasts to determine the optimized concentration. Subsequently, this selected concentration range was further refined in a second concentration range. The chosen concentration was then employed for subsequent experiments. To validate the melanin synthesis inhibitory function of WVYY and PSLPA, melanin content in keratinocytes was measured alongside the optimized concentration (a and b). A 100 nM alpha-MSH was used as a positive control. All significant differences were indicated with alphabetical letters (p < 0.05). Con: Control; PC: Positive control; W: WVYY; P: PSLPA; Co-treatment: WVYY+PSLPA.

Figure S1. Concentration optimization of WVYY and PSLPA and assessment of melanin content. In the initial concentration range, a serial dilution (5, 10, 50, and 100 µM) of WVYY and PSLPA was applied to human keratinocytes (HaCaT cells) and fibroblasts to determine the optimized concentration. Subsequently, this selected concentration range was further refined in a second concentration range. The chosen concentration was then employed for subsequent experiments. To validate the melanin synthesis inhibitory function of WVYY and PSLPA, melanin content in keratinocytes was measured alongside the optimized concentration (a and b). A 100 nM alpha-MSH was used as a positive control. All significant differences were indicated with alphabetical letters (p < 0.05). Con: Control; PC: Positive control; W: WVYY; P: PSLPA; Co-treatment: WVYY+PSLPA.
